# Comment: Flow-injection analysis—The end of the beginning? Segmented-flow analysis— The beginning of the end?

**DOI:** 10.1155/S1463924683000024

**Published:** 1983

**Authors:** C. Riley, B. F. Rocks

**Affiliations:** School of Engineering and Applied Sciences University of Sussex and Biochemistry Department Royal Sussex County Hospital Brighton BN2 5BE UK


Journal of Automatic Chemistry, Volume 5, Number 1 (January–March 1983), pages 1–3

### Editorial

### Are we making the best use of laboratory computer systems?

In this first issue of the 1983 volume of the *Journal of Automatic Chemistry* we can look back on a very successful first year with our new publisher, Taylor & Francis Ltd. Their experience in scientific publishing has been of considerable value to me and the editorial team. We now have a well-produced, accepted and well-presented product. In 1983 we hope to build on this foundation and increase the readership still further. In some senses 1982 was a transition year. Many people will have had subscriptions based on a four-issue basis rather than an annual basis. In 1982 this has been rationalized and all subscriptions are now on an annual basis.

In 1982 much of our editorial matter centred on the use of computers in the laboratory, be it clinical or industrial. The application of computers is a very difficult subject to deal with adequately since the range of experience within the readership is so diverse. What is commonplace for one group is beyond the experience of many others. However, we have attempted in our editorial policy to cater for this and to present detailed examples of practical applications. We will continue to pursue this policy. Should any reader have specific problems relating to interfacing and developing software, we will endeavour to answer such questions in our pages. Within the editorial team we have a wealth of practical experience in applications and in the teaching aspects involved.

Despite the fact that computers and microcomputers are becoming commonplace in our lives, we continue to hear stories of difficulties being experienced implementing computer systems or, indeed, abject failures of systems installed to meet specific needs. In a guest editorial in the *Journal of Clinical Laboratory Automation* (Vol. 2, No. 6) Professor D. M. Block summarizes a series of failures, for a variety of reasons, and warns us to be extremely wary of the so-called 'expert computer consultant'. He tells us 'Be wary of those who teach the art and science of laboratory computing' and, when we are told something by these experts, to question upon what actual personal experience of laboratory computing they are basing their opinions. These are wise words indeed. However, what Professor Block does not suggest is an alternative approach which does not cause so many problems.

Having been through the various stages of defining, specifying, and implementing a laboratory system, I can perhaps add some points which can be of value. Firstly, the various computer experts may well be experts in the use of computers, but it is unlikely that they will have any experience of the particular laboratory for which they are consulting, nor will they have any concept of the staff structure and qualifications of the staff. Secondly, it is vitally important that the person who specifies the final system and justifies its choice and purchase must also be the person who has the responsibility to install the system. Choices made by committees without the need to make the system work rarely come to a good conclusive ending.

The main problem in any system study, be it completely computer or any automation instrumentation, is to find a complete and precise specification of the requirements. This should outline the needs of the organization, its staff, and its customers jointly. At this stage it should not make any attempt to suggest how to solve the problem outlined in the specification. In order to get to this stage correctly, the staff involved must make a real attempt to understand what can and cannot be achieved using computers. Exactly how difficult something is to achieve by computer also has to be realized. Specialized software is an extremely expensive commodity. A detailed specification must also make allowance for one extremely important variable: chance. Specifications often relate to a period of time—implementation and procurement themselves also take time—and the result of this is often that the system is subsequently installed to meet a rather different requirement. However, the difficulty in any specification is to be able to cover as many of these eventualities as possible. A good system, properly specified and installed, will meet many of the precise aims set out in the specification as originally outlined, but in addition will almost always provide additional benefits as a bonus. Without the full involvement and commitment of the users and scientists from the outset, and throughout the many stages involved in the introduction of computer systems into the laboratory, it will be difficult to make full use of the computer.

Hopefully, the many papers which we publish will be of direct help to new computer users. It is, however, a great pity that many of the pitfalls involved are not highlighted in the literature so that new users do not have to re-invent the wheel each time. Computers offer many advantages if properly used—it is correct to be wary of them but, given a good knowledge of their uses, advantages, and disadvantages, a lot can be gained by everyone in the laboratory situation. The scientist must help to identify the needs correctly so that the computers are used wisely, both within laboratories and by instrument companies alike.

Best wishes for successful computing in 1983.

**Peter B. Stockwell**
*Editor*

### Comments

### Flow-injection analysis—The end of the beginning? Segmented-flow analysis—The beginning of the end?

Although several reviews of flow-injection analysis (FIA) have been published [1–4], it is clear from recent remarks made in this *Journal* by Holy [5] that the technique is still not properly understood. We should therefore like to take this opportunity of restating some of the essential features of FIA and of replying to some of the criticisms raised in that paper. We have an advantage over Holy in that we are not committed to any particular mode of automatic analysis. Proponents of FIA may draw comfort from the fact that the Technicon Corporation originally adopted a similar stance with regard to discrete analysis but now actually market two discrete analysers.

As Holy points out, analytical chemists have been pumping unsegmented liquids along tubes for a very long time. However, it has required the perception of workers such as Růžička and Hansen [6] and Stewart *et al.* [7] for the realization that this apparently simple operation was capable of a remarkable degree of exploitation. It was, in fact, the prolific work of Růžička and Hansen [1 and 2] that led us to investigate the potential of FIA in clinical chemistry. Using simple systems constructed from items of equipment commonly used in clinical analyses, we have been able to carry out analyses with economy, speed and accuracy and with negligible carry-over. For those familiar with continuous-flow analysis, the speed of FIA is startling: with fast reactions the analysis may be complete 10 s after introduction of the sample.

Holy suggests that there is a need in FIA for turbulent flow in which the detector may recognize the sample as a square wave. Apart from the difficulty of achieving turbulent flow, it is problematical whether or not flow injection would work effectively under such conditions. Mixing of the sample slug with the carrier stream of reagent depends on the fact that it moves under conditions of laminar flow. The slug adopts a hollow bullet shape and travels along a boundary layer which forms a constantly replenished source of reagent. With the narrow-bore tubing in use, radial diffusion of reagent into slug is extremely rapid. These conditions would not be obtained with turbulent flow.

Holy's commentary [5] contains a number of other misleading statements regarding the practice of FIA. Contrary to his assertions, we find from our own experience and by recourse to the literature that FIA calibration curves are usually linear, that triplicate (or duplicate) measurements are unnecessary and that separation steps (when necessary) can be carried out at least as fast as when using segmented-flow analysis (SFA).

If a single-channel FIA system is compared with a comparable SFA system for carrying out an assay in which the sample reacts rapidly with reagent, it will be evident that:

(*a*) The FIA system will be ready for use almost immediately, whilst the SFA system requires several minutes of operation before its base-line is established.
(*b*) The FIA peaks will be available first—usually within 30 s of sample injection, as against some minutes for SFA.
(*c*) The FIA system will consume less reagent and normally less sample (even after allowing for the small volume which is needed to wash the valve through). The technique known as merging zones [8], in which slugs of both sample and reagent are borne by carrier streams of distilled water and merged downstream of the pump, provides the ultimate in economy. No segmented-flow system can begin to match this.

Clearly, with these fast reactions the FIA approach is much superior; fortunately a number of the most commonly requested tests in clinical chemistry involve such fast reactions.

For many other tests in the clinical field, kinetic assay techniques are preferred. A unique feature of the non-segmented approach is that the flow can be stopped and the sample zone arrested in the cuvette whilst the reaction progresses. The rate of reaction can thus be measured. This cannot be achieved with SFA because the elasticity of the compressed gas in the bubbles will cause movement to continue after the pump is stopped.

In the case of end-point analyses in which relatively long incubations are required (for example, several minutes), SFA systems are still preferable since they offer a faster rate of analysis than can be achieved with FIA. However, in clinical chemistry laboratories such analyses are nowadays relatively rare.

In practice, the weakest link in FIA has been the need to use valves to introduce the sample into the carrier stream. These require some waste of sample and in our hands have been prone to develop leaks. Recently we have developed a number of fully automatic valve-less machines in which there is no waste of sample [9]. These machines can be left on standby indefinitely, they make use of merging zones and they can, when desired, be operated in the stopped-flow kinetic mode.

We have successfully run some 20 different clinical chemistry analyses at 150 samples/h on these machines.

Clinical chemists are beginning to demand selective multichannel analysers which, in contrast to the traditional profile machines, carry out only those tests specifically requested. Between demands these new machines remain on standby. Selective machines so far available have all been based on discrete analysis, but an FIA system such as the one we describe would lend itself perfectly to operation in the selective mode. Because it cannot be operated intermittently there is no place for SFA in this type of machine.

Holy argues that the vast number of SFA machines sold over the last 25 years proves the excellence of the system. The Model T Ford sold in vast numbers in its day, but this is not an argument to condemn the modern motor-car. SFA had the field to itself when it was introduced; the manufacturers of FIA machines, on the other hand, face well-entrenched opposition from established machines and a steadily deepening recession.

### References

1. Růžička, J. and Hansen, E., *Analytica Chimica Acta*, **114** (1980), 19.

2. Růžička, J. and Hansen, E., *Flow Injection Analysis* (Wiley-Interscience, New York, 1981).

3. Ranger, C., *Analytical Chemistry*, **53** (1981), 20a.

4. Rocks, B. and Riley, C., *Clinical Chemistry*, **28** (1982), 409.

5. Holy, H. W., *Journal of Automatic Chemistry*, **4** (1982), 111.

6. Růžička, J. and Hansen, E., *Analytica Chimica Acta*, **78** (1975), 145.

7. Stewart, K. K., Beecher, G. R. and Hare, P. E., *Analytical Biochemistry*, **70** (1976), 167.

8. Bergamin, H. F°., Zagatto, E. A. G., Krug, F. J. and Reis, B. F., *Analytica Chimica Acta*, **101** (1978), 17.

9. Riley, C., Aslett, L. H., Rocks, B. F., Sherwood, R. A., Watson, J. D. McK. and Morgon, J., *Clinical Chemistry* (in press).

**C. Riley and B. F. Rocks**

*School of Engineering and Applied Sciences, University of Sussex and Biochemistry Department, Royal Sussex County Hospital, Brighton BN2 5BE, UK*

### Comments on 'Flow-injection analysis—an idea incomplete?'

This commentary addresses the many inaccuracies and innuendoes which appeared in a recent article by H. W. Holy [1]. In his article, Holy states that flow-injection analysis (FIA) has very limited acceptance and impact 'since commercial instruments have been available since 1959'. The fact is that mass-produced FIA instruments and *significant* marketing efforts for these instruments began around 1978. Thus, it is a bit early to be discussing 'impact' and 'acceptance'. Holy further states that the goal of FIA is 'achievable—theoretically'. The rather large

